# Development of an assessment strategy in preclinical fixed prosthodontics course using virtual assessment software—Part 2

**DOI:** 10.1002/cre2.110

**Published:** 2018-05-31

**Authors:** Ramtin Sadid‐Zadeh, Amin Nasehi, Elaine Davis, Anastasia Katsavochristou

**Affiliations:** ^1^ Department of Restorative Dentistry University at Buffalo School of Dental Medicine New York USA; ^2^ Stony Brook University School of Dental Medicine New York USA; ^3^ Department of Oral Diagnosis University at Buffalo School of Dental Medicine New York USA

**Keywords:** Compare software, dental education, faculty assessment, rubrics

## Abstract

The purpose of this study was to evaluate interrater agreement between faculty and virtual assessments of preparations for complete coverage restorations in preclinical fixed prosthodontics. Teeth prepared during preclinical fixed prosthodontics practical exams at the University at Buffalo School of Dental Medicine were used in this study. Teeth were prepared for fabrication of complete cast, metal ceramic, and all ceramic crowns. The specimens were digitized using an intraoral scanner. Then, they were virtually superimposed on the corresponding standard preparations using Compare software. The software was used to quantify comparison percentages, average finish line widths, and average axial wall heights. Two calibrated faculty members assessed preparations for occlusal/incisal reduction, finish line location, axial wall height, and finish line width using traditional assessment forms. Cohen's kappa coefficient was used to measure interrater agreement between faculty and virtual assessments. Kappa interrater agreement scores ranged between 0.83 and 0.88 for virtually assessed comparison percentages and sums of faculty‐assessed occlusal/incisal reduction and finish line location. Kappa interrater agreement score ranges were 0.64–0.94 and 0.74–0.89 for comparisons of virtual and faculty assessments for axial wall height and finish line width, respectively. Virtual assessments are similar to faculty assessments for occlusal/incisal reduction, finish line location, axial wall height, and finish line width in fixed prosthodontics and can be used as equivalent evaluations of student performance for these criteria.

## 1 INTRODUCTION

Dental education entails not only theoretical training but also practical training in a simulated environment. Practice in simulations laboratory allows students to develop the manual dexterity and technical skills needed for dentistry prior to their exposure to the more challenging patient care environment (Clancy, Lindquist, Palik, & Johnson, 2002). In the U.S.‐accredited dental education model, simulated training is provided during the first 2 years of dental school.

Historically, the initial simulated training consisted of practicing on bench‐top dentiforms, models of jaws bearing replaceable ivory teeth. However, the need for a more realistic simulation laboratory led to the development of contemporary manikins and stations. This newer setting is believed to increase the learning experience of the students prior to applying the technique in the patient care environment (Perry, Bridges, & Burrow, 2015). In addition, over the last decade, advances in technology have facilitated incorporation of virtual reality and three‐dimensional haptic systems into medical and dental training to increase the learning experience of the students (Bongers, van Hove, Stassen, Dankelman, & Schreuder, 2015; Jasinevicius, Landers, Nelson, & Urbankova, 2004; Larsen, Oestergaard, Ottesen, & Soerensen, 2012). However, the validity and value of virtual reality‐based education in dentistry has not yet been fully assessed (Buchanan, 2004).

In addition to the importance of the preclinical simulation environment in dental education, accurate and consistent feedback from faculty is a critical aspect of the educational experience. It is crucial that students receive consistent feedback so that they can use the assessment to improve their performance. However, variations in grading scales, faculty calibration, and subjective faculty assessment can diminish the consistency and value of feedback (Feil & Gatti, 1993). In order to promote more reliable and accurate faculty assessment, the Commission on Dental Accreditation mandates incorporation of assessment forms and faculty calibration for U.S. dental schools (American Dental Association, 2006). However, despite these improvements, multiple studies have shown that faculty interrater and intrarater assessments are not consistent when evaluating dental student performance (Lilley, Bruggen Cate, Holloway, Holt, & Start, 1968; Fuller, 1972; Salvendy, Hinton, Ferguson, & Cunningham, 1973; Sharaf, AbdelAziz, & MEl Meligy, 2007).

Virtual assessment software was proposed as a mechanism to remove faculty‐based subjective error from dental student assessments by providing an objective means of evaluation (Schiff, Salvendy, Root, Ferguson, & Cunningham, 1975; Renne et al., 2013). In support of the idea, calculation of comparison percentage (Comparison%) by virtual assessment software was shown to increase the objectivity and reliability of student assessment in the simulated laboratory setting (Renne et al., 2013). However, Comparison% does not take into consideration the principles of tooth preparation, such as axial wall height (AWH) and total occlusal convergence, when evaluating student performance (Renne et al., 2013). In addition, the validity of the use of Comparison% to assess preparation for complete coverage restorations has been questioned (Callan, Haywood, Cooper, Furness, & Looney, 2015).

In Part 1 of this study, rubrics were developed for evaluating the preparation of complete coverage restorations in the preclinical fixed prosthodontics. Following the virtual quantitative assessment, students utilize Compare software (E4D Technologies, Richardson, TX, USA) to assess their preparations against standard tooth preparations including average AWH, average finish line width (FLW), occlusal/incisal reduction (O/IR), and finish line location (FLL).

Presently, there is no consensus regarding the correlation of virtual quantitative assessments with evaluations from highly trained professionals in the field. Careful evaluation of these correlations is needed to universally establish computerized evaluation as a viable educational tool. The purpose of this study was to verify the virtual assessment rubrics developed in Part 1 of this study. We aimed to evaluate the level of concordance between faculty and virtual assessments for O/IR, FLL, AWH, and FLW in fixed prosthodontics.

## 2 METHODS

This study included 505 collected teeth (Kilgore International Inc., Coldwater, MI, USA) from the class of 2017 at the University at Buffalo School of Dental Medicine (UB SDM). Traditionally, teeth prepared by dental students during their preclinical practical exams are collected and kept for documentation at UB SDM. Specimens used in this study included collected teeth from practical exams for preparation of complete coverage restorations with different preparation designs. Below, the fixed prosthodontics syllabus used for the class of 2017 at UB SDM is described. Then, assessment techniques used to evaluate prepared teeth for this study are defined.

### 2.1 Fixed prosthodontics syllabus at UB SDM

This section describes topics taught to the class of 2017, methods which were used to educate and evaluate students, and educational environment where the course took place. Starting with the class of 2017, the course syllabus for fixed prosthodontics was modified after it was presented and approved by the curriculum committee at UB SDM. Traditionally, students were trained by student–faculty interaction following rubrics assessed by the faculty. However, the class of 2017 was trained by student–faculty and student–Compare software (E4D Technologies, Richardson, TX, USA) interactions following rubrics developed in Part 1 of this manuscript.

Students were trained to use intraoral scanner (Planmeca Corp., Helsinki, Finland) and Compare software (E4D Technologies, Richardson, TX, USA) as part of their course. A faculty member prepared standard preparations taught during the course. Standard preparations were made following the criteria presented to students (Table 1). Then, standard preparations were recorded using an intraoral scanner (Planmeca Corp., Helsinki, Finland). During the academic calendar, students used the three‐dimensional images recorded from standard preparations for self‐evaluation of their performance. The self‐evaluation was done by superimposing of the student preparation against the respective standard preparation in Compare software (E4D Technologies, Richardson, TX, USA). Then, students followed rubrics developed in Part 1 of this manuscript to compare their performances against standard preparations.

**Table 1:** Overview of the design and amount of reduction for each preparation (complete cast crown [CCC], metal ceramic crown [MCC], and all ceramic crown [ACC])

Preparation	Amount of reduction
CCC 46	Chamfer finish line width ≈0.5 mm, functional cusp ≈1.5 mm, nonfunctional cusp ≈1 mm
MCC 24	Lingual chamfer finish line width ≈0.5 mm, buccal shoulder finish line width ≈1.2 mm, functional and nonfunctional cusps ≈2 mm
MCC 46	Lingual chamfer finish line width ≈ 0.5 mm, buccal chamfer finish line width ≈0.8 mm, functional cusp ≈2 mm, nonfunctional cusp ≈1 mm
ACC 21	Modified shoulder finish line width ≈1 mm, incisal edge reduction ≈1.5 mm, lingual reduction ≈1.5 mm
CCC 37	Chamfer finish line width ≈0.5 mm, functional cusp ≈1.5 mm, nonfunctional cusp ≈1 mm
MCC 35	Lingual chamfer finish line width ≈0.5 mm, buccal shoulder finish line width ≈1.2 mm, functional cusp ≈1.5–2 mm and nonfunctional cusps ≈1 mm

As a part of the syllabus, students took practical exams for preparation of complete cast crown of tooth no. 46 (CCC 46), the metal‐ceramic crowns of teeth nos. 24 and 46 (MCC 24 and MCC 46), the all‐ceramic crown of tooth no. 21 (ACC 21), and partial fixed dental prosthesis with the abutment of teeth nos. 37 and 35 (CCC 37, MCC 35). Exams were performed in the UB SDM simulation laboratory where a Kilgore dentiform (Series Model 200, Nissan Dental Products, Japan) was used on a mounted pole to teach preclinical fixed prosthodontics skills. Then, preparations were digitized using an intraoral scanner (Planmeca Corp., Helsinki, Finland) and virtually superimposed on their respective standard preparation using Compare software (Planmeca/E4D Technologies, Richardson, TX, USA). Finally, students were graded for the amount of O/IR and FLL, average of FLW, and average of AWH using Compare software and following rubrics developed in Part 1 of this manuscript. In addition, faculty members used traditional rubrics to assess student's performance for total occlusal convergence, finish of the preparation, quality of the finish line, and adjacent teeth.

### 2.2 Assessment techniques

For the purpose of this study, one operator digitized collected teeth from the above‐mentioned practical exams using an intraoral scanner (Planmeca Corp., Helsinki, Finland). The scans were virtually superimposed on their respective standard preparation using Compare software (Planmeca/E4D Technologies, Richardson, TX, USA). Margin, axial wall base, and occlusal table for each preparation were defined in Compare software. The software was then used to quantify average FLW and AWH. For the anterior tooth, the midlingual AWH was measured instead of the average AWH. In addition, superimposed student and standard preparations were used to quantify Comparison% at 300‐, 350‐, and 400‐μm tolerances. The numeric values extracted from the software were then used to score each preparation based on the ranges for Comparison%, average FLW and AWH from the virtual assessment rubrics developed at UB SDM and presented in Part 1 of this manuscript. Each preparation was scored as excellent (E), standard (S), or standard not met (N) for each criterion.

In addition to virtual assessments, two independent and calibrated faculty members quantified the amount of O/IR, the FLL, AWH, and FLW using traditional assessment forms. The faculty members were not aware of the result of the virtual assessment. Then, each preparation was scored as E, S, or N for the stated criteria. For discordant scores, the faculty members reviewed the preparations following the traditional rubrics until reaching a unified decision. O/IR was quantified using a reduction guide and a periodontal probe. Reduction guides were fabricated on corresponding unprepared teeth using polyvinyl siloxan (Virtual XD, Ivoclar Vivadent, Amherst, NY, USA) and sectioned vertically into one or three slices. Molar reduction guides were sectioned in three locations: at the distolingual cusp tip, the lingual groove, and the mesiolingual cusp tip. Premolar and anterior reduction guides were vertically sectioned on the cusp tip and the mid‐incisal edge, respectively. The amount of O/IR reduction was then measured at each slice using a periodontal probe. FLL and AWH were also assessed using a periodontal probe. FLW was quantified using the corresponding bur for the finish line design and a periodontal probe.

### 2.3 Statistical analysis

Cohen's kappa coefficient (Viera & Garrett, 2005) was used to measure interrater agreement between faculty and virtual assessments of O/IR, FLL, AWH, and FLW (Table 2). In order to evaluate concurrence between Comparison% and faculty assessments, the sum of faculty assessments for O/IR and FLL were compared with Comparison%. The score for the sum of O/IR and FLL was defined as the lowest grade for either criterion in the faculty assessment. For example, for a preparation with FLL scored as N and O/IR scored as E, the sum was considered to be N.

**Table 2:** Alignment of criteria from faculty and virtual assessments

Faculty assessment	Compare software assessment
Occlusal/incisal reduction + finish line location	Comparison percentage
Finish line width	Finish line width average
Axial wall height	Axial wall height average (for anterior tooth, midlingual axial wall height)

## 3 RESULTS

A total of 505 preparations for MCC 24 (*n* = 90), CCC 46 (*n* = 84), ACC 21 (*n* = 84), MCC 46 (*n* = 83), MCC 35 (*n* = 82), and CCC 37 (n = 82) were evaluated in this study. The number of specimens decreased to 82, because eight students left the program during the academic year. Table 3 shows Cohen's kappa coefficient values for comparison between faculty and virtual assessments for Comparison%, AWH, and FLW.

**Table 3:** Cohen's kappa coefficient values for comparisons of virtual and faculty assessments for each criterion

Virtual assessment	Faculty assessment	MCC 24	CCC 46	ACC 21	MCC 46	CCC 37	MCC 35
Comparison% (300 μM)	O/IR + FLL	0.24	0.24	0.24	0.18	0.27	0.18
Comparison% (350 μM)	O/IR + FLL	0.53	0.53	0.69	0.52	0.64	0.52
Comparison% (400 μM)	O/IR + FLL	0.84	0.84	0.83	0.88	0.88	0.86
Average AWH	AWH	0.64	0.64	0.00^a^	0.94	0.86	0.88
Average FLW	FLW	0.79	0.79	0.84	0.74	0.89	0.89

*Note*. ACC = all ceramic crown; AWH = axial wall height; CCC = complete cast crown; FLL = finish line location; FLW = finish line width; MCC = metal ceramic crown; O/IR = occlusal/incisal reduction.

a No coefficients were computed because AWH was identical between the two assessments.

For all preparation designs, interrater agreement was almost perfect (kappa > 0.81) between Comparison% calculated at 400‐μm tolerance and the sum of faculty assessment of O/IR and FLL. Table 4 shows the distribution of E, S, and N scores for Comparison% at 400‐μm tolerance and the corresponding faculty assessments. For preparations scored as N based on Comparison% at 400‐μm tolerance, the majority of faculty assessments were N for either O/IR or FLL or both. Only 0.4% of the preparations (2 of 505) were graded S based on the Comparison% at 400‐μm tolerance but had a faculty assessment of N for either the amount of O/IR or FLL. Similarly, only 4.4% (22 of 505) of the preparations were graded E for Comparison% at 400‐μm tolerance but were scored S for either the amount of O/IR or FLL by the faculty. One preparation (0.2%) was graded E by faculty for both O/IR and FLL but had a virtual assessment score based on Comparison% at 400‐μm tolerance of S.

**Table 4:** Distribution of E, S, and N scores for Comparison% at 400‐μm tolerance and sums of faculty assessments of O/IR and FLL

	MCC 24			CCC 46			ACC 21			MCC 46			CCC 37			MCC 35		
	E	S	N	E	S	N	E	S	N	E	S	N	E	S	N	E	S	N
O/IR + FLL	40	35	15	59	21	4	43	34	7	48	29	6	41	37	4	25	42	15
Comparison%	39	36	15	62	18	4	47	30	7	53	24	6	46	32	4	30	39	13

*Note*. ACC = all ceramic crown; CCC = complete cast crown; FLL = finish line location; MCC = metal ceramic crown; O/IR = occlusal/incisal reduction.

Interrater agreement for FLW was also almost perfect (kappa > 0.81) for ACC 21, CCC 37, and MCC 35. Faculty and virtual assessments of FLW also showed substantial concordance (0.61 ≤ kappa ≤ 0.80) for MCC 24, MCC 46, and CCC 46. Table 5 summarizes the distribution of E, S, and N scores assigned by faculty and virtual assessments for FLW. Only 2.4% of preparations (12 of 505) were scored as N by faculty and S by virtual assessment of FLW. Similarly, 1.2% of preparations (6 of 505) were graded S by the faculty and E using virtual assessment.

**Table 5:** Distribution of E, S, and N scores for faculty and virtual assessments of FLW

Finnish line width	MCC 24			CCC 46			ACC 21			MCC 46			CCC 37			MCC 35		
	E	S	N	E	S	N	E	S	N	E	S	N	E	S	N	E	S	N
Faculty assessment	48	30	12	42	34	8	69	15	0	58	17	8	42	36	4	37	33	12
Virtual assessment	48	30	12	43	36	5	68	16	0	62	18	3	43	36	3	37	36	9

*Note*. ACC = all ceramic crown; CCC = complete cast crown; FLW = finish line width; MCC = metal ceramic crown.

Interrater agreement for AWH was almost perfect (kappa > 0.81) for ACC 21, CCC 37, and MCC 35. Substantial concordance was also observed for MCC 24, MCC 46, and CCC 46, which had kappa values of 0.79, 0.74, and 0.79, respectively. Table 6 depicts the distribution of E, S, and N scores assigned by faculty and virtual assessments of AWH. Only 1.8% of preparations (9 of 505) were scored as E for AWH by faculty members but were assigned a score of S by virtual assessment.

**Table 6:** Distribution of E, S, and N scores for faculty and virtual assessments of AWH (midlingual AWH for anterior tooth)

Axial wall height	MCC 24			CCC 46			ACC 21			MCC 46			CCC 37			MCC 35		
	E	S	N	E	S	N	E	S	N	E	S	N	E	S	N	E	S	N
Faculty assessment	85	5	0	79	5	0	82	2	0	56	27	0	42	36	4	67	14	1
Virtual assessment	80	10	0	78	6	0	82	2	0	54	29	0	43	36	3	65	17	0

*Note*. ACC = all ceramic crown; AWH = axial wall height; CCC = complete cast crown; MCC = metal ceramic crown.

## 4 DISCUSSION

Comparison% was calculated for each student preparation by comparing it with a corresponding standard preparation with a tolerance of 300, 350, or 400 μm. These values were then compared with the combined faculty assessments for O/IR and FLL. At a 400‐μm tolerance, interrater agreement between faculty assessment and Comparison% was almost perfect (kappa > 0.81) for all preparation designs. Only a single preparation (0.2%) was scored E by faculty for the sum of O/IR and FLL and had a virtual assessment of S based on Comparison% at 400‐μm tolerance. This discordant score might be due to the complex anatomy and surface area associated with tooth preparation.

At a 400‐μm tolerance, 4.8% of preparations (24 of 505) received a higher score from virtual assessment compared with faculty assessment. The difference might arise from the methods faculty employ to assess O/IR and FLL. Periodontal probes and reduction guides are used to measure the amount of O/IR. This type of scoring varies based on the location of the section and estimates the remainder of the occlusal reduction because the periodontal probe cannot be used at locations distant from the cut. Because this assessment technique does not consider the overall amount of O/IR, a preparation might be scored as overreduced or underreduced at the slice point, although the overall preparation might have an appropriate amount of reduction. In contrast, Comparison% uses the entire area of the preparation, including the entire occlusal table, to calculate the percentage of matched areas. Moreover, Comparison% calculated by Compare software (Planmeca/E4D Technologies) consistently assesses student work with no subjectivity (Renne et al., 2013). In contrast, when a calibrated faculty member evaluates the same work on separate occasions, they may assign different scores each time (Lilley et al., 1968; Fuller, 1972; Salvendy et al., 1973).

The results suggest that Comparison% at 400‐μm tolerance could be used as an indirect measure of the amount of O/IR and FLL. However, an educator should be aware that the focus on Comparison% could hamper students' understanding of the distinct concepts of O/IR and FLL. The authors suggest that the software could be improved by adding specific measurements for O/IR and FLL. These measurements could be generated by calculating the average FLL for the prepared tooth. Compare software is capable of defining gingival margin. If the software developers were to incorporate a calculation of average finish line height from the gingival margin, this average might be an indicator of FLL. Similarly, a calculation of the average amount of O/IR compared with the unprepared tooth or Comparison% based only on the amount of O/IR after defining the occlusal table could serve as an indicator of the amount of O/IR.

In addition to Comparison%, the average FLW was calculated for each preparation using Compare software, and it was compared with faculty assessment for FLW. Interrater agreement was almost perfect or substantially in agreement (kappa ≥ 0.61) for all preparation designs for this criterion. Virtual assessment of FLW resulted in a higher score compared with faculty assessments for 3.6% of preparations (18 of 505). Twelve of 18 of the inflated scores were graded as N for FLW by faculty but scored as S by virtual assessment. These inflated virtual assessment scores were observed in the CCC 46, MCC 46, MCC 35, and CCC 37 preparations.

The average FLW calculation in the Compare software is dependent on the finish line and axial wall base defined in the software. The axial wall base is defined as the junction between the axial wall and the finish line. It is impotent to know that the axial wall location is not clear for the chamfer finish line design. For CCC 37 and CCC 46, this discordance might be due to the difficulty of defining the axial wall base for a chamfer finish line design using the software. For MCC 35 and MCC 46, in addition to the abovementioned explanation, two different finish line designs were prepared for the teeth, which could have influenced the grading, resulting in inflation. In order to improve assessment of FLW, the authors suggest incorporation of automated processes to define the finish line and axial wall using the software. This step would minimize user variation in defining these lines. In addition, when a preparation has two different finish line designs, the average FLW may not be instructive for students, especially regarding the FLW required for each finish line design. Therefore, allowing separate calculations for the average of the two finish line designs on a preparation could be beneficial from an educational perspective.

The average AWH was also calculated for each preparation using the software and scored in faculty assessments. Interrater agreement for AWH was almost perfect or substantially in agreement (kappa ≥ 0.61) for all preparation designs. Even those preparations with only substantial interrater agreement had kappa values (0.79, 0.74, and 0.79) approaching the almost perfect range. Virtual assessment of AWH yielded nine preparations (1.8%) with lower grades compared with the faculty assessments (S instead of E). These differences may have resulted from specific features of the interproximal AWH, where the finish line is anatomically located more occlusally compared with the finish line of the buccal and lingual surfaces. In addition, after preparation of a tooth for full‐coverage restoration, the interproximal occlusal table is anatomically located more gingivally compared with the buccal and lingual occlusal table. These two factors result in a shorter interproximal AWH compared with the buccal and lingual AWH. This shorter interproximal AWH might account for the small percentage of lower scores. Furthermore, the horizontal components of a masticatory cycle and parafunctional habits exert forces on a full‐coverage crown that are customarily faciolingual in direction (Goodacre, Campagni, & Aquilino, 2001). As a result, faculty assessments may focus primarily on the facial and lingual AWH, causing them to ignore or miss measurement of the interproximal AWH.

## 5 CONCLUSIONS

Within the limitations of this study, the following conclusions can be drawn:

1. Virtual assessment of the Comparison% at a tolerance of 400 μm can be used to evaluate O/IR and FLL.
2. Interrater agreement between the Comparison% at a 400‐μm tolerance and the sum of faculty‐assessed O/IR and FLL is almost perfect (kappa > 0.81) for all preparation designs. However, virtual assessment may be associated with slight inflation in grading.
3. Interrater agreement between virtual and faculty assessment of FLW was almost perfect or substantially in agreement (kappa ≥ 0.61) for all preparation designs. However, virtual assessment of FLW may be associated with grade inflation.
4. Interrater agreement between virtual and faculty assessment of AWH was almost perfect or substantially in agreement (kappa ≥ 0.61) for all preparation designs. However, virtual assessment of AWH was associated with a lower grade in 1.8% of student preparations.
